# Myofibroblasts persist through immune privilege mechanisms to mediate oral submucous fibrosis: Uncovering the pathogenesis

**DOI:** 10.1016/j.jobcr.2024.10.008

**Published:** 2024-10-20

**Authors:** Mohit Sharma, Smitha Sammith Shetty, Sonal Soi, Raghu Radhakrishnan

**Affiliations:** a Department of Oral Pathology, SGT Dental College Hospital & Research Institute, Gurugram, Haryana, 122505, India; b Department of Oral Pathology, Manipal College of Dental Sciences, Manipal, Manipal Academy of Higher Education, Manipal, 576104, India; c Department of Conservative Dentistry and Endodontics, Manav Rachna Dental College, Faridabad, India; d Academic Unit of Oral and Maxillofacial Pathology, School of Clinical Dentistry, University of Sheffield, Sheffield, S10 2TA, UK

**Keywords:** Myofibroblast, Fas-FasL pathway, PD1-PD-L1 pathway, Immune checkpoint protein, Tregs, Oral submucous fibrosis

## Abstract

Immune privilege is the ability to tolerate foreign antigens without eliciting an inflammatory immune response. Several mechanisms explain a structure's immune privilege status, which is regulated by innate and adaptive immune responses. The role of myofibroblasts in perpetuating fibrosis by acquiring an immune privileged phenotype against the backdrop of oral submucous fibrosis (OSF) is evolving. Myofibroblasts persist through the Fas/FasL autocrine pathway and induce apoptosis in epithelial cells, explaining the juxtaposition of apoptotic cells in areas of fibrosis. However, increased matrix stiffness, in addition to activating TGF-β, reduces Fas surface expression in myofibroblasts, increasing their resistance to apoptosis. The reciprocal amplification loop between the immune checkpoint proteins programmed death-ligand 1 (PD-L1) and TGF-β involves the YAP-TAZ and SMAD2,3 pathways and dramatically enhances profibrotic signalling. Increased matrix stiffness also enhances cMYC expression, which subsequently amplifies PD-L1 levels on myofibroblasts. The increase in PD-L1 on the myofibroblast microengineers the phenotype of CD4^+^ T cells homing to fibrotic areas by acting on the programmed cell death protein 1 (PD-1) receptor on the T-cell surface, converting these cells from antifibrotic cells to profibrotic cells that produce IL-17A and TGF-β. This manuscript provides mechanistic insight into how myofibroblasts avoid apoptosis in OSFs by evading the immune system. Targeting an immune-privileged phenotype in myofibroblasts with FAS-FASL pathway-dependent characteristics is an ideal strategy for reversing OSF.

## Graphical abstract

**Figure:**
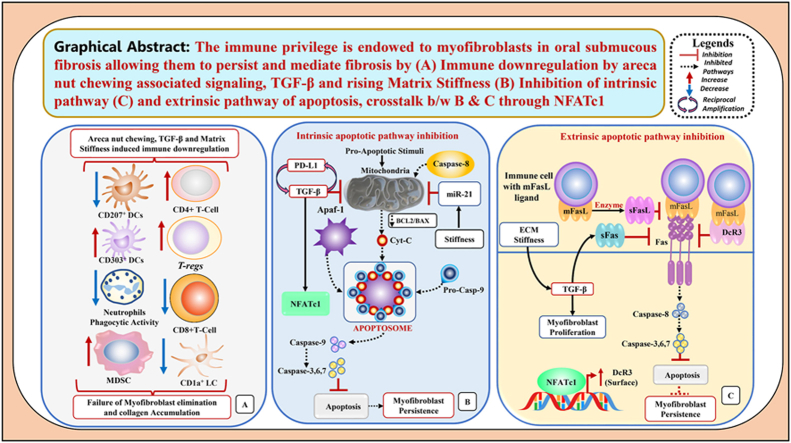

## 1 Introduction

Oral submucous fibrosis (OSF) is an aberrant wound healing response comprising inflammation of the oral mucosa with gradual progressive fibrosis leading to an increased risk for malignant transformation. The main reason for the development of OSF is areca nut consumption, the components of which induce various cellular responses, such as inflammation, oxidative stress, and DNA damage. The change in the molecular profile of OSF from a condition characterized by the disturbed expression of cytokines and growth factors contributes to epithelial atrophy and supports epithelial‒mesenchymal transition (EMT), which increases the risk of cancer. Due to the high prevalence of OSF in South Asia, particularly in India, the incidence rates are high. Therefore, raising awareness about OSF and facilitating effective interventions is urgently needed for individuals and communities.^1^, ^2^, ^3^

Fibroblasts, which are quiescent cells, are responsible for normal turnover of the extracellular matrix (ECM). Any injury to tissue causes the differentiation of fibroblasts into myofibroblasts. The contractile property of myofibroblasts results in accelerated wound closure. During routine wound healing, activated myofibroblasts are primed for apoptosis, thus promoting faster wound closure. However, myofibroblasts tend to evade apoptosis, leading to their uninterrupted accumulation under fibrotic conditions.^4^ Nonhealing wounds in cancerous tissue and excessive fibrous healing in fibrosis are characterized by the occurrence of cancer-associated fibroblasts (CAFs) and the persistence of myofibroblasts, respectively.^3^^,^^5^, ^6^, ^7^ While CAFs play an essential role in modulating the malignant phenotype, myofibroblasts are central to the development of fibrosis.

Immune privilege (IP) is the ability to endure foreign antigens without eliciting a viable inflammatory immune response. The IP is an evolutionary adaptation to protect vital structures with limited repair capacity from collateral inflammatory immune damage. Tissue grafts survive longer without rejection at immune-privileged sites. Corneal and knee meniscal transplantation takes advantage of the IP status of the transplantation sites.^8^^,^^9^ Some immune-privileged sites in the body include the anterior chamber of the eye, testes, ovaries, articular cartilage, pregnant uterus, central nervous system, placenta, fetus, heart valves, bone marrow, hair follicle, retina, and even tumor cells.^8^^,^^10^, ^11^, ^12^, ^13^, ^14^, ^15^, ^16^

Immune privilege (IP) can also be viewed as a form of immunological tolerance, where it allows harmful invaders like viruses and parasites to persist for extended periods, even though they would normally be eliminated by the organism's robust immune system.^17^ We believe that this mechanism may enable the persistence of certain organelles in cells beyond their necessary function, such as the continued presence of myofibroblasts after completing tissue repair.

While researching references on immune privilege (IP), we found several articles suggesting that the IP state may not be confined to traditional IP sites but could also apply more broadly to myofibroblasts.^18^, ^19^, ^20^, ^21^, ^22^, ^23^ Further research prompted us to apply this concept to OSF myofibroblasts and their potential acquisition of the IP state, which appears to be a pivotal mechanism driving their accumulation and the fibrotic progression in OSF. Despite the abundant literature on OSF, the reasons for the persistence of myofibroblasts, the key contributors to progressive and irreversible fibrosis, remain insufficiently understood. This review aims to bridge that gap by exploring how the IP state contributes to the survival and persistence of myofibroblasts in OSF. By deepening the understanding of disease pathogenesis through the lens of the IP state of myofibroblasts, the review seeks to spur research into innovative therapeutic targets to restrict the fibrotic progression of OSF.

## 2 Immune evasion by cancer cells and myofibroblasts in fibrotic disorders

The FAS gene in humans encodes a protein known by various names: Fas receptor (FasR), Fas, cluster of differentiation 95 (CD95), or apoptosis antigen 1 (APO-1). This receptor is a death receptor located on the cell surface that triggers apoptosis when it binds to Fas ligand (FasL).^24^, ^25^, ^26^ FasL/CD95L/CD178 is a 37-kDa type II transmembrane protein (mFasL) found on the surface of immune cells. It binds to its receptor Fas and triggers apoptosis. FasL induces apoptosis in Fas-expressing cells through direct cell-to-cell contact.^25^^,^^27^^,^^28^

mFasL^+^ lymphocytes induce apoptosis in cancer cells by interacting with the surface Fas receptor on cancer cells. However, cancer cells can resist apoptosis from mFasL^+^ immune cells, particularly lymphocytes, by expressing a soluble form of FasL (sFasL). This soluble sFasL competes with mFasL to bind to the Fas death receptor on the cancer cell surface, allowing cancer cells to evade the immune system and accumulate in tissues.^29^^,^^30^ Thus, cancer cells exhibit IP properties and can evade the immune response.

In a recent study of oral squamous cell carcinoma (OSCC) and oral leukoplakia patients, it was revealed that tryptophan 2,3-dioxygenase (TDO2)-positive myofibroblasts (TDO2^+^ myofibroblasts) are located near the tumor nest and attract CD4^+^ T cells and CD8^+^ T cells. These TDO^2+^ myofibroblasts promote the conversion of CD4^+^ T cells into regulatory T cells (T-regs) and CD8^+^ T cells into exhausted T cells by coexpressing immune checkpoint inhibitors such as T-cell immunoglobulin and mucin domain-containing protein 3 (TIM-3) and programmed cell death protein 1 (PD-1).^31^, ^32^, ^33^, ^34^

PD-1, a member of the immunoglobulin superfamily, binds to its ligand, programmed death-ligand 1 (PD-L1), preventing T lymphocyte activation, homing, and destruction of harmful cells such as cancerous cells. In malignancies, antigen-specific T lymphocytes exhibit increased PD-1 expression, which is upregulated through Smad3 signaling mediated by TGF-β1. This pathway allows cancer cells to evade immune surveillance by inhibiting effective T cell responses.^35^

One study examined PD-L1/PD-1 expression in OSCC patients with and without OSF. PD-L1 expression was significantly higher in OSCC patients with OSF and strongly correlated with lymph node metastasis and advanced tumor stages. High PD-L1 expression was associated with worse outcomes in these patients. The study suggested that PD-L1 could serve as an unfavorable prognostic indicator, and targeting the PD-L1/PD-1 signaling pathway might represent a promising therapeutic strategy, particularly for OSCC patients with OSF.^36^ Thus, there is evident myofibroblast-mediated immune downregulation in OSCC patients, and a similar mechanism might also be involved in oral fibrosis.

Postinjury, the differentiation of myofibroblasts and immune cell-induced apoptosis of myofibroblasts upon conclusion repair prevent excessive accumulation of ECM.^4^ However, in a fibrotic disorder, myofibroblasts persist and accumulate further through immune evasion.^18^^,^^25^^,^^37^^,^^38^ The ability of myofibroblasts to persist and thereby evade the immune system is unique to fibrotic myofibroblasts but not regular myofibroblasts.^18^^,^^25^^,^^36^^,^^38^ It could thus be hypothesized that myofibroblasts persist and accumulate in OSF due to the acquisition of an IP phenotype.^39^

## 3 Mechanism of IP in cells and tissues

Immune-privileged cells (IPCs) are the key component cells of tissues and organs with immune privilege (IP). Several mechanisms have been identified that protect these tissues from immune attacks, particularly from cytotoxic T lymphocytes (CTLs)/CD8^+^ T cells, which are essential for the resolution of fibrosis.^40^, ^41^, ^42^ These protective mechanisms ensure that IPCs can evade immune responses, contributing to the persistence of fibrosis in certain conditions.^43^^,^^44^

a) Major histocompatibility complex (MHC) class Ia molecules are typically absent in IPCs, protecting them from lysis by CTLs.^42^
b) However, the loss of MHC class 1a molecules increases the susceptibility of IPCs to lysis by natural killer (NK) cells. IPCs circumvent susceptibility to NK cell lysis by expressing nonclassical MHC class 1b molecules such as HLA-G and HLA-E, which interact with NK inhibitory receptors to stall NK cell-mediated lysis. Additionally, the IP sites and organs express the soluble form of HLA-G, which causes the apoptosis of CTLs.^40^
c) The IP sites and organs express the apoptosis-inducing ligand FasL and tumor necrosis factor-related apoptosis-inducing ligand (TRAIL), which induce apoptosis in infiltrating immune cells, such as neutrophils, macrophages, and activated T cells.^40^
d) As the complement activation pathway supplements the immune system, IPCs and tissues evade this pathway by increasing the expression of surface molecules such as decay-accelerating factor and membrane cofactor protein, which inhibit complement pathways.^42^^,^^45^
e) IPCs and tissues also secrete complement regulatory proteins, which inactivate complement pathways.^40^^,^^45^
f) IPCs and tissues secrete immunosuppressive cytokine-like TGF-β, which suppresses NK cell-mediated cytolysis.^19^
g) The recruitment of immunosuppressive regulatory T cells (Tregs), a specialized population of T cells that can inhibit the T-cell population and suppress the immune response.^19^^,^^41^
h) The production of neuropeptides suppresses inflammation by preventing macrophage activation.^46^
i) Immune privileged organs contain myeloid cell-derived lineages such as immunosuppressive DCs that express indoleamine 2,3-dioxygenase (IDO-2,3), especially CD303^+^ plasmacytoid DCs.^47^, ^48^, ^49^ IDO-2,3 suppresses the effector function of CTLs, depletes tryptophan and induces T-cell apoptosis.^49^
j) Basic fibroblast growth factor (bFGF) enhances immune tolerance in allogeneic cartilage transplants by strengthening the function of Tregs. This action helps to create immune tolerance, shielding the implanted cartilage from immune system attacks and promoting the long-term survival of the tissue.^14^

These mechanisms serve as broad templates to support the hypothesis that myofibroblasts possess an IP phenotype in the pathogenesis of OSF.

## 4 Mechanisms of development of the IP phenotype in OSF

The means through which myofibroblasts accumulate in OSF and acquire an IP phenotype are based on two plausible mechanisms. (1) Role of the immunosuppressive environment induced by TGF-β, the PD-L1‒PD-1 pathway, Treg differentiation, and NK-cell downregulation. (2) The role of the FAS-FasL and TRAIL apoptotic pathways in myofibroblasts was validated via IP. Owing to the paucity of literature, other mechanisms have not been investigated.

### 4.1 Development of an immunosuppressive environment in OSF

**Fig. 1:**
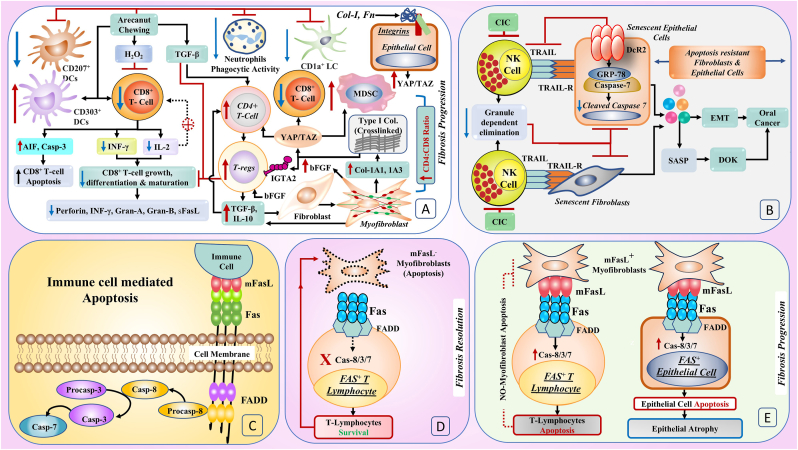
The mechanism leading to the immune privilege of myofibroblasts in OSF **1A.** Areca nut-induced downregulation of CD8^+^ T-cell growth, differentiation, and maturation through H_2_O_2_ leads to the suppression of INF-γ, a reduction in IL-2 (this reduction leads to feedback suppression of CD8^+^ T cells), and an increase in AIF and Casp-3, which leads to an increase in CD8^+^ T-cell apoptosis. TGF-β further promotes the differentiation of CD4^+^ T cells into T-regs, which produce more TGF-β. TGF-β causes myofibroblastic differentiation and the production of Col-1A1 and Col-1A3. Upon crosslinking, this collagen activates the Integrin Subunit Alpha 2 (IGTA-2) receptor on T-regs to stimulate them to produce even more TGF-β. Areca nut chewing downregulates the immune system by suppressing CD1a^+^ Langerhans cells, reducing neutrophil phagocytic activity, reducing CD207^+^ dendritic cells, and increasing the number of immunosuppressive CD303^+^ DCs. The downregulation of CD8^+^ T cells and upregulation of CD4^+^ T cells lead to a high CD4^+^CD8^+^ T-cell ratio with fibrosis progression. The ECM-induced YAP-TAZ pathway in epithelial cells results in the upregulation of Tregs and MDSCs and the downregulation of CD8^+^ T cells, leading to the upregulation of fibrosis and malignancy. **1B.** The circulating immunocomplex (CIC) found in OSF patients suppresses the NK cell-mediated elimination of senescent epithelial cells and senescent fibroblasts through the TRAIL-TRAIL-R interaction. This also results in reduced NK cell granule-dependent elimination of senescent epithelial cells and senescent fibroblasts. The upregulation of DcR2 in senescent epithelial cells also inhibits the NK-mediated elimination of senescent fibroblasts through the TRAIL-TRAIL-R interaction. This also results in epithelial and fibroblast accumulation and the promotion of fibrosis. The intracellular cascade initiated by DcR2 in senescent epithelial cells leads to the formation of the GRP78‒Caspase-7 complex, resulting in reduced cleaved caspase-7 and subsequent resistance to apoptosis. The combined SASP from senescent epithelial cells and senescent fibroblasts promotes the EMT and malignant transformation of DOKs. **1C.** Prototypic model of immune cell-mediated elimination through the apoptosis of unwanted cells through the FasL-Fas interaction. **1D.** FasL^−^ Myofibroblasts cannot drive Fas^+^ CD8^+^ cell apoptosis through the Fas/FADD/Cas-8/3/7 pathway but instead undergo apoptosis, which is the typical path of fibrosis resolution. **1E.** As myofibroblasts acquire immune privilege, they start expressing FasL and induce the apoptosis of CD8^+^ T cells, allowing their persistence in fibrotic tissues. These FasL^+^ myofibroblasts induce apoptosis in the Fas^+^ epithelium through the Fas/FADD/Cas-8/3/7 pathway, explaining the juxtaposition of the atrophic epithelium with fibrosis in OSF.

The use of areca nuts, which are known to cause OSF, inhibits T-cell activation and interleukin-2 (IL-2) and interferon-γ (INF-γ) production through increased H_2_O_2,_ and this effect is reflected in reduced intracellular glutathione levels in CTLs.^50^^,^^51^ Decreased IL-2 leads to reduced T-cell growth and differentiation and impaired maturation of T-cell subsets.^50^ Areca nut chewing also results in decreased complement- and IgG-mediated neutrophil phagocytosis, which may be due to reduced expression of complement receptors and Fc receptors and reduced F-actin production.^52^ TGF-β, the master regulator of fibrosis, accelerates myofibroblast conversion and reduces the activity of CTLs by decreasing the secretion of FasL/soluble FasL (sFasL), INF-γ, Granzyme-A (Gran-A), Granzyme-B (Gran-B), and perforin^53^ (Fig. 1A). Haque et al. (2001) reported that INF-γ is reduced with OSF progression and that its restitution prevents OSF.^54^^,^^55^

TGF-β promotes the differentiation of CD4^+^ T cells into immunosuppressive Tregs,^56^ which in turn produce greater quantities of TGF-β and IL-10.^56^ These cytokines act on fibroblasts, converting them into myofibroblasts, which further amplify the levels of TGF-β and IL-10, perpetuating a cycle that promotes fibrosis.^57^, ^58^, ^59^ However, chimeric antigen receptor (CAR) T cells, in response to TGF-β, may not preferentially expand immunosuppressive Treg cells but may instead convert this immunosuppressive cytokine into a potent T-cell stimulant.^60^ Thus, CAR-T-cell therapy could be utilized for the therapeutic control of fibrosis.^58^ Tregs inhibit the autocrine differentiation of CTLs by competing with IL-2,^61^ promoting the secretion of another immunosuppressive cytokine, IL-10. The cross-linking of type I collagen enhances the proliferation of Tregs via the integrin alpha 2 (ITGA2) subunit.^62^^,^^63^ Activated Tregs in fibrotic tissue through TGF-β secretion upregulate COL1A1 and COL3A1 expression in myofibroblasts.^56^^,^^64^^,^^65^ Collagen, fibronectin, and other ECM proteins engage integrins on basal epithelial cells, triggering YAP/TAZ signalling, which then upregulates myeloid-dependent suppressor cells (MDSCs) and Tregs and downregulates CTLs.^66^ OSF fibroblasts can also acquire immune privilege (IP) ability by producing basic fibroblast growth factor (bFGF), which increases the number of Treg cells. (Fig. 1A).^3^^,^^14^ This contributes to an immunosuppressive environment that promotes the progression of fibrosis and malignancy.^59^^,^^66^

NK cells play a role in eliminating senescent cells and preventing fibrosis.^67^^,^^68^ NK cell activity is reduced in oral precancerous lesions^69^ and is the result of circulating immune complexes (CICs) in the blood of OSF patients.^70^ Granule exocytosis, but not death receptor-mediated apoptosis, is required for the NK cell-mediated killing of senescent cells.^68^ Senescent epithelial cells and fibroblasts escape NK cell killing by upregulating decoy receptor 2 (DcR2), attenuating NK-mediated cell death. While DcR2 binds to the ligand TRAIL, it lacks the downstream signalling of the death receptor pathway.^67^ High expression of DcR2 promotes glucose-regulated protein 78 (GRP78)-Caspase 7 complex formation, resulting in decreased cleaved caspase 7 levels and leading to SASP and apoptosis resistance.^71^ Loss of NK cells renders the antisenescence mechanism inactive^67^^,^^69^ and facilitates the accumulation of senescent myofibroblasts in OSF.^4^^,^^72^ The senescence-associated secretory phenotype (SASP) derived from myofibroblasts induces EMT in OSF and causes the conversion of dysplastic oral keratinocytes (DOKs) into cancer cells, facilitating cancer progression (Fig. 1B).^4^^,^^72^

In brief, areca nuts inhibit T-cell function, decrease the phagocytic activity of neutrophils, and promote the differentiation of CD4^+^ T cells into immunosuppressive Tregs. Tregs inhibit the autocrine differentiation of CTLs and secrete the immunosuppressive cytokine IL-10. Areca nut chewing promotes the secretion of pro-apoptotic factors such as AIF and caspase 3 (Casp-3) and induces CTL apoptosis. The loss of NK cells in OSF allows the accumulation of senescent cells, especially myofibroblasts. These findings imply that impaired immunosurveillance is a harbinger of fibrosis in OSF (Fig. 1A and B).

We recently reported a progressive decrease in CD1a^+^ Langerhans cells and CD207^+^ dendritic cells from the control group to leukoplakia, OSF, OSF with OSCC (OSF-OSCC), and OSCC arising de novo.^48^ Compared with those in the control group, a progressive increase in IDO-2,3-expressing plasmacytoid DCs (CD303^+^) was observed in the OSF, OL, OSF-OSCC, and OSCC groups. This suggests a sustained increase in the IP state during progression through these stages.^47^^,^^48^ Our results revealed a sequential decrease in immune surveillance mechanisms with malignant progression through premalignant stages of oral potentially malignant disorders, including OSF (Fig. 1A).

### 4.2 Role of the Fas-FasL apoptotic system

To delve further into this concept, reviewing the Fas-FasL apoptotic pathway is crucial. Upon the engagement of Fas with mFasL trimers, Fas multimerizes through the Fas-associated death domain (FADD), which recruits procaspase and caspase 8 to form a death-inducing signalling complex. In addition to activating each other, the eight units of procaspase-8 activate downstream effector caspases, such as caspases 7 and -3, which mediate apoptosis.^28^ A prototypic model of Fas-FasL interaction-induced apoptosis is shown in Fig. 1C.

As discussed above, the Fas-FasL interaction is used by immune cells to dispose of unwanted cells via apoptosis. FasL^−^ myofibroblasts cannot induce Fas^+^ lymphocyte apoptosis through the caspase pathway. On the other hand, these surviving lymphocytes induce apoptosis in FasL^−^ myofibroblasts, allowing fibrosis resolution (Fig. 1D).^18^

It has been demonstrated in a murine model of bleomycin-induced lung fibrosis that specific immune surveillance of lung myofibroblasts fails because of the emergence of their immune-privileged cell phenotype. In normal regenerating tissue, immunosurveillance mechanisms prevent fibrosis by inducing apoptosis in myofibroblasts (Fig. 1D). However, FasL^+^ lung myofibroblasts in idiopathic pulmonary fibrosis evade immune cell-induced apoptosis, thus allowing their uninterrupted accumulation (Fig. 1E).^20^ In normal oral mucosa, the CD4:CD8 ratio is approximately 1:2.^73^ Areca nuts preferentially promoted the apoptosis of CD8^+^ T cells (Fig. 1A). Additionally, FasL^+^ myofibroblasts induced apoptosis in CD8^+^ T cells (Fig. 1E). This causes an inversion of the typical mucosal CD4:CD8 ratio in OSF tissues, suggesting a possible imbalance in immunoregulation.^74^ A CD4:CD8 ratio of 2.6:1 in OSF indicates a relative increase in CD4^+^ T cells compared with CD8^+^ T cells in OSF tissues compared with normal mucosa.^54^ This ratio is reduced to 2.0:1 upon treatment with INF-γ,^54^ which acts directly on CD8^+^ T cells to increase their numbers (Fig. 1A).^75^ The higher CD4^+^CD8^+^ T-cell ratio in OSFs after areca nut use and its reversal following antifibrotic treatment is due to subdued immunosurveillance in OSFs.

Epithelial injury and apoptosis are central to the development of fibrosis. While activated mFasL^+^ lymphocytes are the main inducers of apoptosis in epithelial cells,^76^^,^^77^ they undergo apoptosis via myofibroblasts. However, despite coexpressing Fas and mFasL, mFasL ^+^ macrophages^77^ or epithelial cells do not undergo apoptosis via either autocrine or paracrine signalling.^77^ This finding was confirmed in a bleomycin-induced lung fibrosis mouse model, demonstrating that only mFasL^+^ myofibroblasts are capable of orchestrating the apoptosis of Fas^+^ lung epithelial cells, both in vitro and in vivo.^25^^,^^77^ A similar mechanism explains why the atrophic epithelium in OSF is caused by juxtaepithelial hyalinization (Fig. 1E).^77^^,^^78^

### 4.3 Co-option of the intrinsic apoptosis pathway in the acquiring IP myofibroblast phenotype

**Fig. 2:**
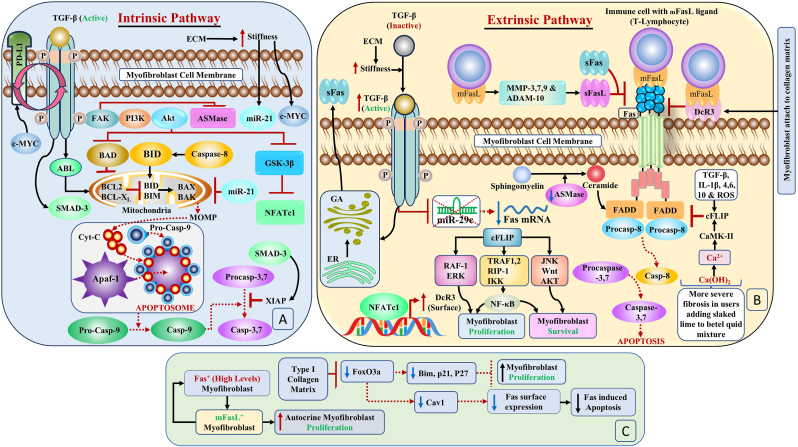
**Further mechanisms of immune privilege of myofibroblasts in OSF** **2A. Co-option of the Intrinsic Pathway of Apoptosis** Apoptosis, triggered by intracellular death stimuli, induces mitochondrial outer membrane permeabilization (MOMP), leading to mitochondrial swelling, rupture, and the release of proapoptotic proteins. The effectors of MOMP include BCL-associated X protein (BAX) and BCL-2 homologous antagonist/killer (BAK), which are activated by BH3-interacting domain death agonist (BID) and Bcl-2-interacting mediator (BIM). Through activation of the Abelson murine leukemia (ABL) pathway, TGF-β blocks intrinsic apoptosis in myofibroblasts by upregulating BCL-2 and Bcl-xL. TGF-β can act downstream of the Casp-9 pathway to inhibit apoptosis through SMAD-3-induced XIAP. Myofibroblasts express an immune checkpoint protein called programmed death ligand 1 (PD-L1), which promotes TGF-β-dependent ECM deposition and cell migration. T cells express the cell surface receptor programmed cell death protein 1 (PD-1), which, in interaction with PD-L1 on myofibroblasts, prevents homing T lymphocytes from destroying other cells, such as cancerous cells and myofibroblasts. **2B. Co-option of the Extrinsic Pathway of Apoptosis** Increased fibrosis, because of stiffness, leads to the activation of TGF-β and the downregulation of Fas surface expression. Fas surface expression was also reduced through the downregulation of miR29C. These mechanisms explain the persistence of myofibroblasts due to their immune privilege in fibrosing tissues. More severe fibrosis in users who added slaked lime to the betel quid mixture can be explained by the upregulation of cFlip. cFlip is also upregulated by TGF-β, IL-4, IL-6, IL-10, IL-1β, and ROS. The upregulation of cFlip through the formation of the multimolecular complex of RAF1, TRAF1,2, and RIP-1 promotes the proliferation of myofibroblasts through the ERK and NF-κB pathways and promotes myofibroblast survival through the JNK, Wnt, and AKT pathways. This signalling by cFLIP also converts the Fas apoptotic signal on myofibroblasts into autocrine proliferative signalling. The soluble form of FasL, the sFasL generated by the actions of MMP-3,7,9 and ADAM-10 on mFasL and sFas, upregulates TGF-β and induces apoptosis resistance in myofibroblasts. Through TGF-β_1_, ET/Akt/GSK-3β/NFATc1 (nuclear), membrane decoy receptor 3 (DcR3) is upregulated. DcR3 binds to FasL and inhibits apoptosis. This antiapoptotic effect is amplified by the binding of myofibroblasts to the collagen matrix through DcR3 upregulation. **2C**. The accumulation of the type I collagen matrix downregulates FoxO3a, which in turn promotes myofibroblast proliferation through Bim, p21, and P27 downregulation. FoxO3a reduction also leads to reduced Cav-1 levels, which, in turn, reduce Fas levels and cause Fas-induced apoptosis. High Fas^+^ levels on myofibroblasts interact with high mFasL^+^ levels on neighboring myofibroblasts to promote autocrine myofibroblast proliferation.

The intrinsic pathway of apoptosis, triggered by intracellular death stimuli such as DNA damage, oxidative stress, or oncogene activation, induces mitochondrial outer membrane permeabilization (MOMP), leading to mitochondrial swelling, rupture, and the release of proapoptotic proteins such as cytochrome C (Cyt C) in the cytosol.^4^ Once in the cytosol, Cyt C allosterically activates protease-activating factor 1 (Apaf 1), Apaf-1 binds to Cyt C, and procaspase-9 (Procasp-9) forms an apoptosome, which then activates Procasp-9 to form Caspase 9 (Casp-9).^4^^,^^79^^,^^80^ Casp-9 acts on Pro-Caspases 3 and 7 and converts them to Caspases 3 and 7 (Casps 3 and 7) as the final step of apoptosis. However, Casp 3 and 7 can be inhibited by X-linked inhibitor of apoptosis protein (XIAP), which stalls the apoptosis program (Fig. 2A).^4^

The effectors of MOMP are BCL-associated X protein (BAX) and BCL-2 homologous antagonist/killer (BAK), which are activated by BH3-interacting domain death agonist (BID) and Bcl-2-interacting mediator (BIM). BID and BIM inhibit B-cell lymphoma-2 (BCL-2) and B-cell lymphoma extralarge (Bcl-xL).^4^

Through activation of the Abelson murine leukemia (ABL) pathway, TGF-β blocks the intrinsic apoptotic pathway in myofibroblasts by upregulating BCL-2 and Bcl-xL. Additionally, TGF-β_1_ obstructs the inherent pathway by blocking the FAK–PI3K–AKT signalling pathway, inhibiting the proapoptotic protein BCL-2-associated death promoter (BAD). Caspase 8, which is derived from an extrinsic pathway, induces apoptosis by inducing BID.^4^ TGF-β can act downstream of the Casp-9 pathway to inhibit apoptosis through SMAD-3-induced XIAP. In mouse models of systemic sclerosis, targeted silencing of XIAP decreased WNT-induced fibroblast activation and collagen release.^81^

On their surface, myofibroblasts express PD-L1, which acts as a brake to control the body's immune reactions by acting on immune cells carrying the PD-1 receptor.^21^^,^^59^ Although PD-L1 serves as a ligand for PD-1, it can, through reverse signalling, inhibit TGF-β_2_R protein degradation and TGF-β_1_R mRNA degradation, thereby increasing TGF-β signalling.^22^ TGF-β upregulates PD‐L1 expression in fibroblasts in a SMAD-2/3- and YAP-TAZ-dependent manner.^21^^,^^82^ Hence, a reciprocal amplification loop exists between the PD-L1 and TGF-β pathways.^22^^,^^82^ Silencing of PD‐L1 signalling hinders the TGFβ-dependent induction of ECM deposition and cell migration.^82^ PD‐L1 has also been shown to act as a SMAD3 cofactor to initiate α-SMA transcription, thus promoting fibroblast transdifferentiation into myofibroblasts.^21^ The oncogene cMYC has been shown to upregulate PD-L1 in cells and may act as an immune privilege enabler oncogene in OSF. Additionally, cMYC is upregulated in OSF due to increased matrix stiffness, which is sensed by β1 integrin (Fig. 2A).^39^^,^^83^

PD-1^+^CD4^+^ T cells were found in three different fibrosis models, i.e., idiopathic pulmonary fibrosis (IPF), sarcoidosis, and bleomycin. These cells exhibited decreased proliferation and elevated TGF-β/IL-17A expression. The significant CD4^+^ T-cell fraction that expressed TGF-β was composed of PD-1+ Th17 cells. Collagen-1 is produced when PD-1+CD4^+^ T cells and human lung fibroblasts are cocultured.^21^

MicroRNA miR-21 is induced by ECM stiffness, and it increases BCL-2 expression, decreases BAX expression, and promotes the survival of stiffness-activated myofibroblasts. After being mechanically stimulated on pathological scar-stiff polymer substrates, miR-21 preserves the contractile phenotype of myofibroblasts even weeks after these cells are relocated to the soft substrate; this phenomenon is referred to as “mechanical memory.” (Fig. 2A).^4^

### 4.4 Co-option of the extrinsic apoptosis pathway for acquiring the IP phenotype

Myofibroblast accumulation in tissues leads to increased ECM deposition, increased matrix stiffness and activation of TGF-β. Both stiffness and increased TGF-β levels resulting from fibrosis inhibit surface Fas in myofibroblasts, contributing to their immune privilege. Increased stiffness and activated TGF-β inhibit surface Fas through the downregulation of miR-29C, the antifibrotic miRNA responsible for the upregulation of Fas,^76^ thus protecting myofibroblasts from Fas-induced apoptosis. TGF-β_1_-mediated FAK signalling blocks the extrinsic pathway of apoptosis by inhibiting acid sphingomyelinase (ASMase), an enzyme that facilitates FAS-mediated apoptosis by generating ceramide from sphingomyelin (Fig. 2B).^4^

Fibrotic myofibroblasts resist apoptosis through the upregulation of cellular FLICE (FADD-like IL-1β-converting enzyme)-inhibitory protein (c-FLIP), the release of soluble FasL (sFasL), and the reduced expression of Fas transmembrane death domains.^20^^,^^84^^,^^85^ c-Flip is upregulated by calcium hydroxide (slaked lime)/calcium/calmodulin-dependent protein kinase II (CaMKII).^86^ This explains the severity of fibrosis among betel quid users who received slaked lime. c-Flip can be upregulated by TGF-β, IL-4,6,10, 1β, and ROS.^87^ This leads to the upregulation of the Jnk, Wnt, and Akt pathways, promoting myofibroblast survival.^88^ Thus, c-Flip converts proapoptotic signals into myofibroblast survival and proliferative signals. cFlip upregulates a molecular complex of tumor necrosis factor receptor (TNFR)-associated factor-1,2 (TRAF-1,2), receptor-interacting protein 1 (RIP1) and RAF protooncogene serine/threonine-protein kinase 1 (RAF1).^88^ cFlip promotes myofibroblast proliferation by upregulating the TRAF-1,2/RIP1/IκB kinase (IKK)/nuclear factor kappa B (NF-κB) pathway and through the TRAF-1,2/RAF1/ERK signalling pathway (Fig. 2B).^88^

sFasL is generated by the action of MMP-3, 7, 9, and ADAM-10 on mFasL.^27^ sFasL competes with membrane mFasL for binding to Fas. However, sFasL does not induce Fas oligomerization, preventing proapoptotic mFasL signalling.^24^^,^^27^

Myofibroblasts resist apoptosis even after expressing high levels of Fas due to the upregulation of c-FLIP, which changes apoptotic signalling to proliferative signalling in myofibroblasts. The occurrence of more severe fibrosis in users who added slaked lime to the betel quid mixture is explained by the upregulation of c-FLIP by slaked lime (Fig. 2B). sFasL is generated by the action of MMPs on mFasL and competitively inhibits mFasL-Fas-induced apoptosis. sFas also inhibits the apoptotic activity of mFasL. Fas/mFasL interactions form autocrine myofibroblast proliferation loops that promote fibrosis.

Fas/mFasL interactions form autocrine myofibroblast proliferation loops that promote fibrosis in several organs.^18^ Similarly, soluble Fas (sFas) is upregulated by TGF-β and inhibits the apoptotic activity of mFasL (Fig. 2B and C).^76^

A type I collagenous matrix has been shown to promote fibroblast apoptosis.^23^ However, the upregulation of Akt by TGF-β_1_ and endothelin (ET) suppresses glycogen synthase kinase-3β (GSK-3β) by converting it to an inactive form.^4^^,^^89^ GSK-3β suppresses the nuclear factor of activated T cells, cytoplasmic 1 (NFATc1), and its downregulation leads to the nuclear localization of NFATc1.^23^^,^^89^ Nuclear NFATc1, in turn, upregulates membranous decoy receptor 3 (DcR3). DcR3 binds to FasL, suppressing the FasL/Fas pathway in fibroblasts. When myofibroblasts attach to the collagen matrix, DcR3 is further upregulated, amplifying the antiapoptotic potential of myofibroblasts (Fig. 2B).^23^

### 4.5 Miscellaneous pathways conferring the IP state to myofibroblasts

The type I collagenous matrix suppresses FoxO3a; suppresses Bim, p21, and P27; and increases myofibroblast proliferation. The downregulation of FoxO3a leads to reduced caveolin-1 (Cav-1) and reduced Fas expression, inhibiting Fas-induced apoptosis (Fig. 2C).^23^

Type I collagenous matrix-induced fibroblast apoptosis is circumvented by the upregulation of DcR3, which suppresses FasL-induced apoptosis.^90^ The collagenous matrix then upregulates DcR3, amplifying the antiapoptotic potential of myofibroblasts. The type I collagenous matrix suppresses Fas, Bim, p21, and P27 expression, inhibits Fas-induced apoptosis and increases myofibroblast proliferation (Fig. 2C).

## 5 Implications & future research directions

According to the above discussion, myofibroblasts behave as IPCs that escape attack by CTLs. Myofibroblasts induced the apoptosis of Fas^+^ CTLs (Fig. 1E). Targeting various pathways that promote myofibroblast survival may inhibit fibrosis. Although fibrosis is considered irreversible, it has been found otherwise in both mouse and human models of fibrosis.^4^ Drugs that target the extrinsic apoptosis pathway, such as AT-406 and TLY12, are suggested to treat fibrosis.^4^ Drugs that target senescence-like dual therapy with dasatinib and quercetin, ABT263, and GKT137831 have been shown to promote apoptosis in target cells.^4^ Inhibitors of MMP-3, 7, 9, and ADAM10 can reduce the formation of sFasL and cell death resistance in fibroblasts.^27^ The use of neutralizing antibodies against FasL expressed by myofibroblasts can prevent induced apoptosis in T cells.^18^ Brefeldin A is a cytotoxin produced by a fungus that inhibits protein transport from the endoplasmic reticulum to the Golgi, reducing the secretion of profibrotic sFas from fibroblasts in cell culture supernatants.^76^^,^^91^ Another therapeutic avenue is the use of HDAC inhibitors such as vorinostat and droxinostat to inhibit cFlip and its variants.^88^ Another view on the central role of myofibroblasts as IPCs could be linked to the chronic force and tissue tension generated during the chewing of BQ/tobacco. During wound healing, myofibroblasts may serve as coordinating cells to achieve antiapoptotic cellular states in cancer cells and noncancer cells, including immune cells such as CD8^+^ T cells. During all these adaptations at the site of wound healing during the chronic use of chewing BQ/tobacco, chemical compositions are essential, and these chemicals may fuel the metabolic needs of myofibroblasts. Therefore, in addition to slaked lime, other carcinogenic and noncarcinogenic oncometabolites, such as BQ/tobacco, should be considered for possible modulation of the tissue environment from proapoptotic to antiapoptotic by the involvement of myofibroblasts. Oncometabolites derived from BQ/tobacco at the site of chronic wound generation and subsequent wound healing may contribute to the epigenetic modulation of myofibroblasts, increasing the scope of epigenetic inhibitors.

## 6 Conclusion

While quiescent fibroblasts mediate the normal turnover of the ECM, any tissue injury initiates the differentiation of fibroblasts into myofibroblasts, which accelerate wound closure through their contractile property. These activated myofibroblasts undergo apoptosis upon the completion of healing. However, myofibroblasts evade apoptosis under fibrotic conditions, leading to their uninterrupted accumulation. Nonhealing wounds in cancerous tissue and excessive fibrous healing in fibrosis are characterized by the occurrence of cancer-associated fibroblasts (CAFs) and the persistence of myofibroblasts, respectively. While CAFs play an essential role in modulating the malignant phenotype, myofibroblasts are central to the development of fibrosis.

IP is the ability to tolerate foreign antigens without eliciting an inflammatory immune response. Several IP sites exist in the human body, anterior chamber of the eye, testes, ovaries, articular cartilage, pregnant uterus, central nervous system, placenta, fetus, heart valves, bone marrow, hair follicle, retina, and even tumor cells. The mechanisms of IP at these sites were studied and are listed as a template to determine the role of myofibroblasts in perpetuating fibrosis through the acquisition of an IP phenotype in oral submucous fibrosis (OSF) tissues, which was the basis of our hypothesis. Myofibroblasts gain immune privilege through the Fas/FasL autocrine pathway and induce apoptosis in epithelial cells, explaining the juxtaposition of apoptotic cells in areas of fibrosis. Another contributing mechanism is increased matrix stiffness, which, in addition to activating TGF-β, reduces Fas surface expression in myofibroblasts. The molecular pathways leading to acquisition of the immune-privileged phenotype of myofibroblasts in OSF are ideal targets for reversing OSF.

## Patient consent

Not Applicable.

## Ethics approval and consent to participate

Not applicable.

## Availability of data and materials

Not applicable.

## Competing interests

The authors declare that they have no competing interests.

## Authors' contributions

MS, SS and SSS contributed to the conception of the topic and manuscript writing. MS prepared the figures, and RR edited and revised the manuscript.

## Consent for publication

Not applicable.

## Conflict of interest

No conflicts of interest exist.

## Ethical clearance

Not Applicable.

## Funding

This work was supported by the 10.13039/501100009053Wellcome Trust/DBT India Alliance, sanction order No. “IA/CPHI/18/1/503,927″, dated September 27, 2019.

## Source of funding

None.

## Declaration of competing interest

The authors declare that they have no known competing financial interests or personal relationships that could have appeared to influence the work reported in this paper.
